# A new mixed reality tool for training in minimally invasive robotic-assisted surgery

**DOI:** 10.1007/s13755-023-00238-7

**Published:** 2023-08-02

**Authors:** Sergio Casas-Yrurzum, Jesús Gimeno, Pablo Casanova-Salas, Inma García-Pereira, Eva García del Olmo, Antonio Salvador, Ricardo Guijarro, Cristóbal Zaragoza, Marcos Fernández

**Affiliations:** 1 Institute of Robotics and Information Technology and Communication (IRTIC), University of Valencia, Valencia, Spain; 2 General and Gastrointestinal Surgery, Fundación Investigación Consorcio Hospital General Universitario de Valencia (FIHGUV), Valencia, Spain; 3 Thoracic Surgery, Fundación Investigación Consorcio Hospital General Universitario de Valencia (FIHGUV), Valencia, Spain

**Keywords:** Mixed reality, Training, Annotation, Robotic-assisted surgery

## Abstract

Robotic-assisted surgery (RAS) is developing an increasing role in surgical practice. Therefore, it is of the utmost importance to introduce this paradigm into surgical training programs. However, the steep learning curve of RAS remains a problem that hinders the development and widespread use of this surgical paradigm. For this reason, it is important to be able to train surgeons in the use of RAS procedures. RAS involves distinctive features that makes its learning different to other minimally invasive surgical procedures. One of these features is that the surgeons operate using a stereoscopic console. Therefore, it is necessary to perform RAS training stereoscopically. This article presents a mixed-reality (MR) tool for the stereoscopic visualization, annotation and collaborative display of RAS surgical procedures. The tool is an MR application because it can display real stereoscopic content and augment it with virtual elements (annotations) properly registered in 3D and tracked over time. This new tool allows the registration of surgical procedures, teachers (experts) and students (trainees), so that the teacher can share a set of videos with their students, annotate them with virtual information and use a shared virtual pointer with the students. The students can visualize the videos within a web environment using their personal mobile phones or a desktop stereo system. The use of the tool has been assessed by a group of 15 surgeons during a robotic-surgery master’s course. The results show that surgeons consider that this tool can be very useful in RAS training.

## Introduction

Minimally invasive surgery (MIS) represents a fundamental paradigm shift in the field of surgery that has influenced the techniques used in almost every surgical field [1] and caused a re-evaluation of clinical strategies. This type of surgical procedure is based on making small incisions in the patient's body, through which surgeons introduce different instruments and usually a laparoscopic camera. MIS is associated with less pain, shorter hospital stays and fewer complications because, by avoiding large incisions, infection risks are minimized and post-surgical pain is reduced.

A step forward in MIS is robotic-assisted surgery (RAS), also called Robotic Minimally Invasive Surgery (RMIS), or Robotic-Assisted Minimally Invasive Surgery (RAMIS). In this surgical paradigm, the surgeons do not directly handle the surgical tools. Instead, they control a series of robotic arms that are introduced into the patient through small incisions. RAS avoids the surgeon having to stand for a long time, avoids human hand tremor and offers surgeons the possibility of making movements that would be physically impossible if they had to hold the surgical instruments with their own hands, such as twists and full 360° turns. In addition, the surgeon obtains an enhanced perception of the surgical target as stereoscopic cameras are almost always employed in these surgical devices. Of course, the operating consoles have stereoscopic viewers that contribute to increasing the surgeon’s precision and accuracy.

Two major drawbacks have emerged with the introduction of this surgical paradigm [2]: (i) the learning curve of RAS is steep compared to open surgery, or even with respect to endoscopic MIS (i.e., non-robotic MIS); (ii) the increased investment that is needed to use these devices, due to the costs associated with the acquisition and maintenance of robotic equipment and the use of disposable instruments. These two problems are not always compensated by a reduction in complications and shorter hospital stays. Therefore, it is imperative to help reduce these two drawbacks. With respect to the latter, an accurate estimation of the real cost–benefit ratio of RAS is very difficult to obtain [3], but advances in technology—especially in miniaturization and ICT-supported features–, and increased competition are expected to make this paradigm much more affordable and cost-effective. However, we need to tackle the former problem and reduce the steep learning curve of robotic-assisted surgery, since training in RAS is different to training in non-robotic MIS. In fact, it has been recently proven that mastery in laparoscopy is not necessary before initiating robotic surgical training [4], although it could definitely help.

Training in RAS entails two fundamental aspects: skill and knowledge. Regarding the former, skill is usually trained using simulators [5], which allow the surgeon's skills to be polished without putting a patient at risk. As for the latter, a widely used teaching resource is the display of videos of previous RAS procedures, with which RAS trainees (future RAS-certified surgeons) can observe, from a first-person perspective, how to carry out surgical procedures using RAS. Since these types of surgeries are minimally invasive, and the body of the patient is not exposed for trainees to observe the procedure live, videos are an even more important teaching asset.

A very important question in this regard is that surgeons have a stereoscopic view of the tissues while performing the surgical procedure. This is an important difference with respect to most endoscopic surgical procedures, which are usually mono. Thus, when using surgical videos for teaching RAS, these videos should be viewed by surgeons with a stereoscopic display in their training process, so that RAS trainees can observe the procedure as if they were performing it from the control console of the surgical robot itself. Otherwise, the teaching value of the video will be reduced, since the surgeons will not be able to perceive the depth of the tissues as occurs when they use the surgical robot. To this end, special devices such as Virtual Reality glasses or Head-Mounted Displays (HMD) are necessary to allow the correct visualization of the teaching resources. In addition, raw videos need to be edited to remove/conceal uninteresting sections and highlight the key elements with annotative elements.

As a way to help solve this training problem, in this article we present a new tool based on the use of the Mixed Reality (MR) paradigm for the visualization and annotation of stereoscopic videographic material associated with RAS procedures, so that surgeons can use it to explain how they perform a surgical procedure and train future RAS surgeons. The tool is interactive, highly dynamic and collaborative. It works on web technology and low-cost hardware, making it almost universally accessible, and it can reproduce the RAS videos in an MR environment, because the tool allows the surgical videos to be enhanced with virtual annotations, increasing their teaching value.

The term Mixed Reality refers to those applications that allow the creation of environments in which real and virtual objects are combined within a single display. When the amount of digital information is small and used to augment real objects, it is often referred to as Augmented Reality (AR), as in [6–8]. However, when the environment consists solely of digital (virtual) objects, it is called Virtual Reality (VR) instead of MR, as in [9, 10]. Our tool mainly displays real information in the form of video material. However, we augment this information with virtual annotations that are properly registered in 3D with respect to the position of the real content. Furthermore, we display all content in stereoscopic mode, providing a realistic 3D visualization. Hence, the proposed tool can be classified as an MR tool.

## Related work

Unlike the *Fundamentals of Laparoscopic Surgery* (FLS) [11], which is considered the preferred laparoscopic skills curriculum, no single curriculum has yet emerged as the gold standard in the field of robotic surgery [12]. However, a series of key elements can be identified in RAS training, such as robotic skill training, procedure training and bedside (patient-side) training.

**Fig. 1:**
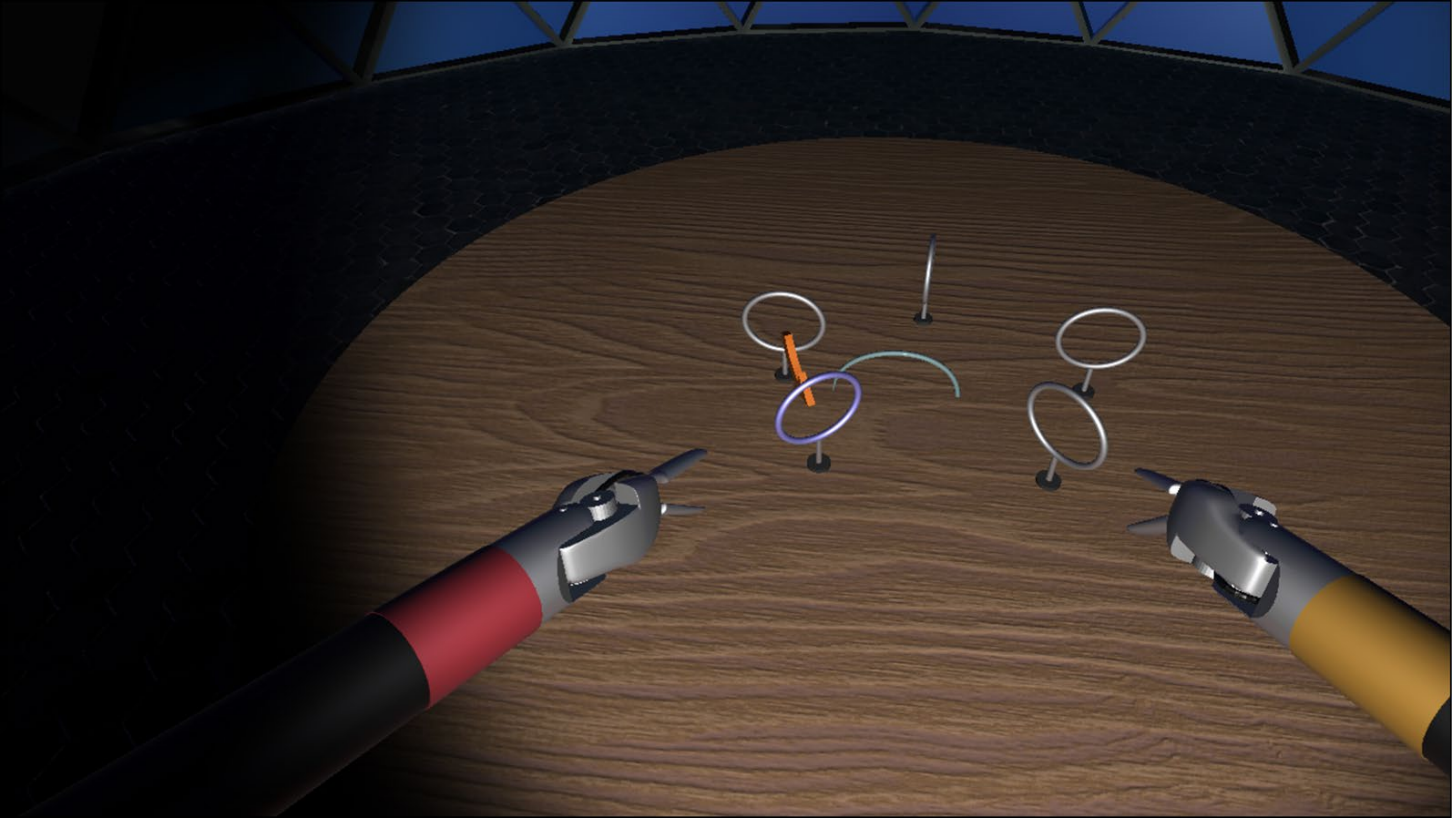
RAS simulator used for skill training, developed for the ViPRAS project (virtual planning for robotic-assisted surgery) (see acknowledgment section). The simulator is utilized to master the use of the different tools available in robotic-assisted operating rooms

Regarding robotic skill training, the use of VR-based simulators to train RAS skills is not uncommon [5, 12, 13]. In fact, VR is a highly appropriate technology for this, since the goal of skill training is *only* to learn how to use the robot console and its tools. Figure 1 shows a snapshot of a RAS simulator, created by the authors using Unity 3D in their laboratory, as part of one of the projects leading to this tool. A similar Unity-based simulator can be found in [14].

Unlike MIS, where haptic feedback is essential—VR is generally unable to provide this important perceptual cue–, the loss of tactile feedback in VR-based RAS training is less of a factor, since current robotic surgery systems do not feature haptics [12]. Therefore, VR can be considered a very useful paradigm for RAS skill training. However, there are other options as well. Skill training also includes dry-lab and wet-lab models that are used as training elements for RAS [15].

Dry-lab models (i.e., inanimate models), such as pelvic dome trainers, are largely used to practice basic skills, such as suturing or dissecting. An interesting proposal in this regard is [16], in which a physical simulator of pulmonary veins is constructed with sensor feedback. Data coming from these sensors are used for objective skill assessment.

In wet-lab models, surgical techniques are trained on cadaveric or live animals and/or on human cadavers [15]. Although all these simulation-based training tools still need competency benchmarks in order to be introduced into existing curricula, and it is still unclear how to translate the acquired skills in simulation into the operating room, it seems clear that these new tools are promising and have the potential to bring about important improvements to the surgical training curriculum [17, 18].

Regarding procedure training, the use of MR or VR in robotic surgery is also not new. There are VR-based simulators that include software modules to train specific surgical procedures, besides basic robotic skills. However, the availability of surgical procedures and the fidelity of the simulation is still limited. Therefore, the use of videos to show real surgical procedures is still important. Extending the analysis to other use cases, the spectrum of applications of MR to RAS is large, with many examples [19]. However, most of these applications are designed for surgical guidance [20, 21], surgery planning [22], port placement [23], or skill training [24]. The use of MR for procedure training is somewhat limited. An example of this could be [25], in which a simulation, combining real 3D videos of a lateral skull base surgery with virtual reproductions of the temporal bone models, is proposed, creating a virtual operating room in which novice surgeons could improve their ability in this complicated surgical procedure. However, the virtual models are not integrated within the video, reducing the immersion of the system.

Bedside training is another important element in surgical training. However, its use in RAS as a training asset, albeit important [12], is limited, since surgical robots often have only one console, and thus, only the lead surgeon is actually experiencing the surgery firsthand. External displays help other surgeons observe the surgical procedure, although with the use of offline videos the procedure can be analyzed in more detail and depth. Dual consoles also exist, but are uncommon and expensive. Therefore, RAS videos are a good solution to watch RAS-based surgical procedures and use them as training assets. However, these videos are stereoscopic and need to be reproduced with stereoscopic displays, which are not always available, unless low-cost solutions are proposed, as we do in the present work. A very interesting approach for bedside training in RAS is the one shown in [26] where an AR application is proposed to aid the patient-side assistant of a Da Vinci surgical robot. The system uses an optical see-through HMD so that the assistant is able to see a virtual representation of the robotic instruments, the laparoscopic field-of-view and the laparoscopic video inside the patient’s body.

Of course, the use of videos as a teaching resource in surgery is not new [27–29], and they have already proven to be a valuable training asset [27]. In fact, they can even be found on social media with positive but also negative consequences [30]. More complex video-based training systems have also been proposed in the surgical field. For instance, [31] shows the use of 360º videos in orthognathic surgery in combination with VR and natural interaction.

There are also several academic works describing the use of stereoscopic videos, as we propose. For instance, in [32] the use of stereoscopic videos as a teaching resource was assessed and was regarded as useful in otolaryngology surgery training by all the participants. There are also some works that focus on how to produce these stereoscopic videos. For instance, [33] presents a method for producing and viewing 3D videos in transoral robotic surgery (TORS) without the acquisition of dedicated 3D recording equipment. They focus on low-cost approaches as we do, although we go much further since we present an MR-based application, an authoring tool, and a collaborative display of stereoscopic RAS videos. A somewhat similar approach can be found in [34], although they focus only on the video capture process of the Da Vinci Xi system, something that has limited academic value. Other similar works also focus on how to acquire stereoscopic videos for surgery [35, 36], whereas we focus on how to broadcast and enhance these videos with virtual information rather than on the acquisition process. A very different approach is presented in [37], which proposes a system that records not only stereo laparoscopic videos, but also stores kinematic data from both surgeon controllers, as well as kinematic data from all instrument arms, in a synchronized manner.

There are also systems designed to perform streaming of stereoscopic videos of robotic surgeries [38], but these works are very different from what we are proposing. Finally, Artificial Intelligence (AI) is also being explored to label or annotate surgical videos [39, 40]. This approach, albeit promising, focuses on how to choose texts rather than how to visualize them, as we do in this research. AI tools also require significant amounts of training data and may require domain-specific training for different surgical areas.

As can be seen, there are several works dealing with the use of stereoscopic RAS videos or with the use of MR in the surgical field. However, there is a research gap when it comes to the use of MR-enhanced stereoscopic videos designed for RAS training. The authors have been unable to find any work that is similar to what we propose. There is also a lack of applications dealing with virtual annotations in stereoscopic RAS videos. Although the use of annotation in the surgical field is also not uncommon [41–47], existing solutions do not work with stereoscopic videos, nor do they show the annotations in MR or perform tracking of the annotated element. Therefore, we believe our approach covers an existing research gap.

## Materials and methods

The tool presented here is a web-based MR application. The use of web technology is justified by the need to provide universal access to the tool, since medical doctors do not want to waste time installing software suits in order to watch their videos. Web access is almost ubiquitous. Therefore, the development of a web-based tool allows teachers (experts) and students (trainees) to use the application in different locations and/or with different computing devices. The use of MR is justified by the need to enhance the videos with virtual information. They need to be shown in 3D, since RAS systems are always used with stereoscopic cameras and stereoscopic consoles. Thus, the addition of virtual 3D elements is a natural step forward. The problem is that the recorded videos lack depth information, and therefore the introduction of virtual elements which can be properly placed within the 3D space of the video is challenging.

Two types of users can be identified in this application: *RAS teachers* and *RAS students*. Both are expected to be surgeons, but the teachers will be expert surgeons, with vast experience in RAS procedures, who want to share their knowledge to other novel surgeons who do not have experience in RAS procedures—or at least have little or no experience in the type of procedure being taught–. Both teachers and students will access the tool by a standard user-password authentication system, but they will have different interfaces. Both types of users will have access to a list of RAS videos with different options. The students can watch the available videos with all the information, including a stereoscopic view. The teachers can do the same, but they can also edit the information shown in the videos and upload new videos. At any time, a collaborative view is possible, where the teacher controls the playback of the video, while the students watch the stereoscopic visualization. In the next sections each interface is described.

### Teacher’s view

**Fig. 2:**
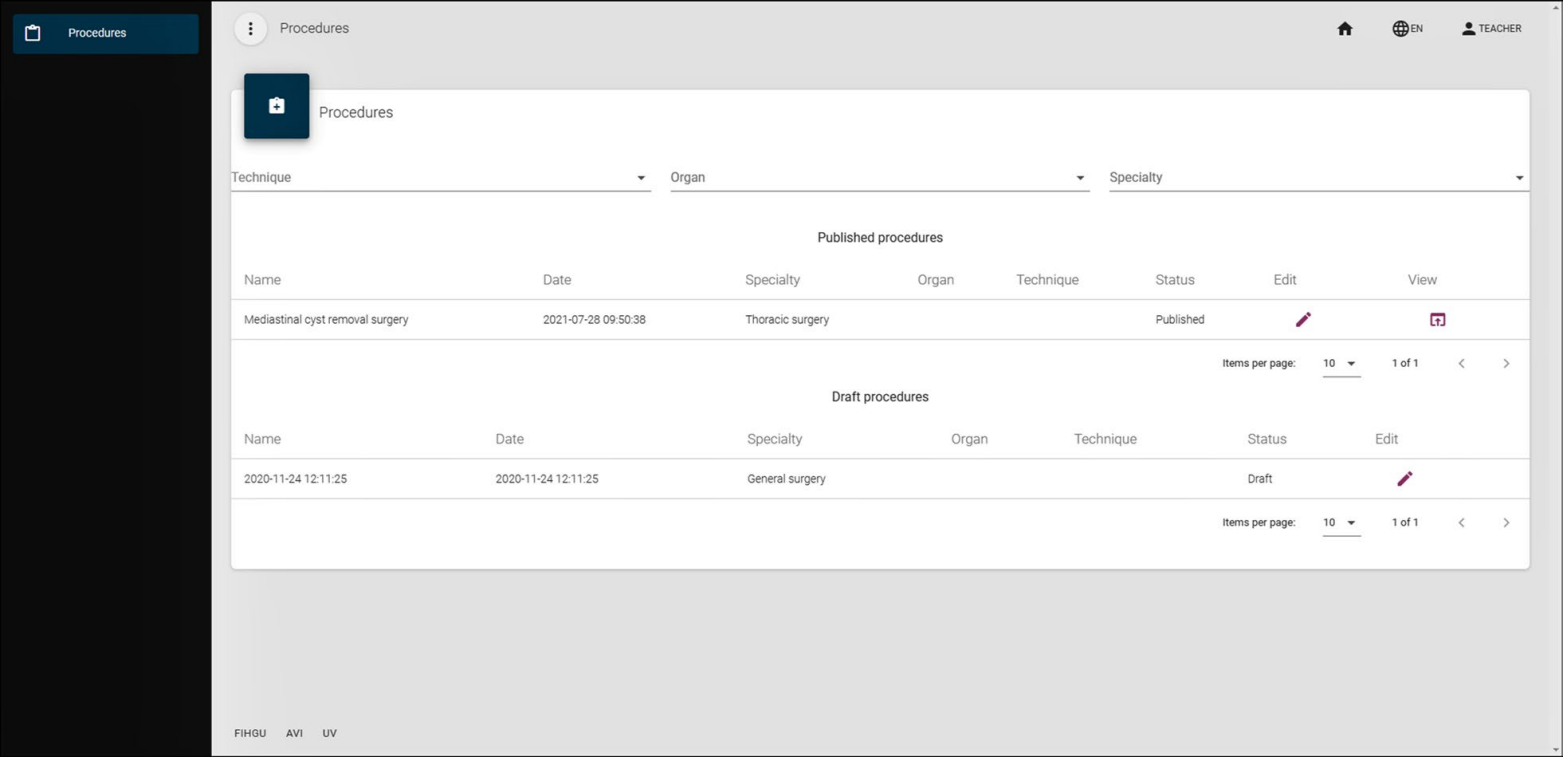
Intervention management interface. Students can access the published interfaces, but do not have access to the authoring tool

Once the teacher has logged in, the tool presents them with the list of surgical procedures that have been stored in the system. New surgical procedures can be added, by simply uploading the stereoscopic video of the surgery. Information about date, surgical specialty, organ and technique can be supplied. Figure 2 shows the interface of this intervention management module. Surgical interventions can be stored in *draft* mode or in *published* mode. The students will not be able to view the interventions that are in draft mode; however, they will be able to view the published interventions. In addition, a collaborative display of the videos is possible. In this latter option, the teacher always decides and controls when the surgical procedure is shown (broadcast) to the students. To this end, each surgical procedure has a display button next to it, which creates a virtual room for students to connect and watch the intervention, enhanced with virtual content. When a room is created, a room code is generated. This code is needed for the students to access the MR-enhanced surgical video.

### Teacher’s authoring tool

Before the teacher shows—using the collaborative display—or allows students to watch—if they choose to watch the videos without the collaborative mode—a surgical video, the teacher needs to have edited it after uploading it. Each surgical procedure in the intervention management interface has an edit button next to it that gives access to a wide set of functionalities to edit the video. This video editor is the authoring part of our application, since it allows the teacher to configure how the video is displayed and how much virtual content will be added to it, in order to create an MR experience.

**Fig. 3:**
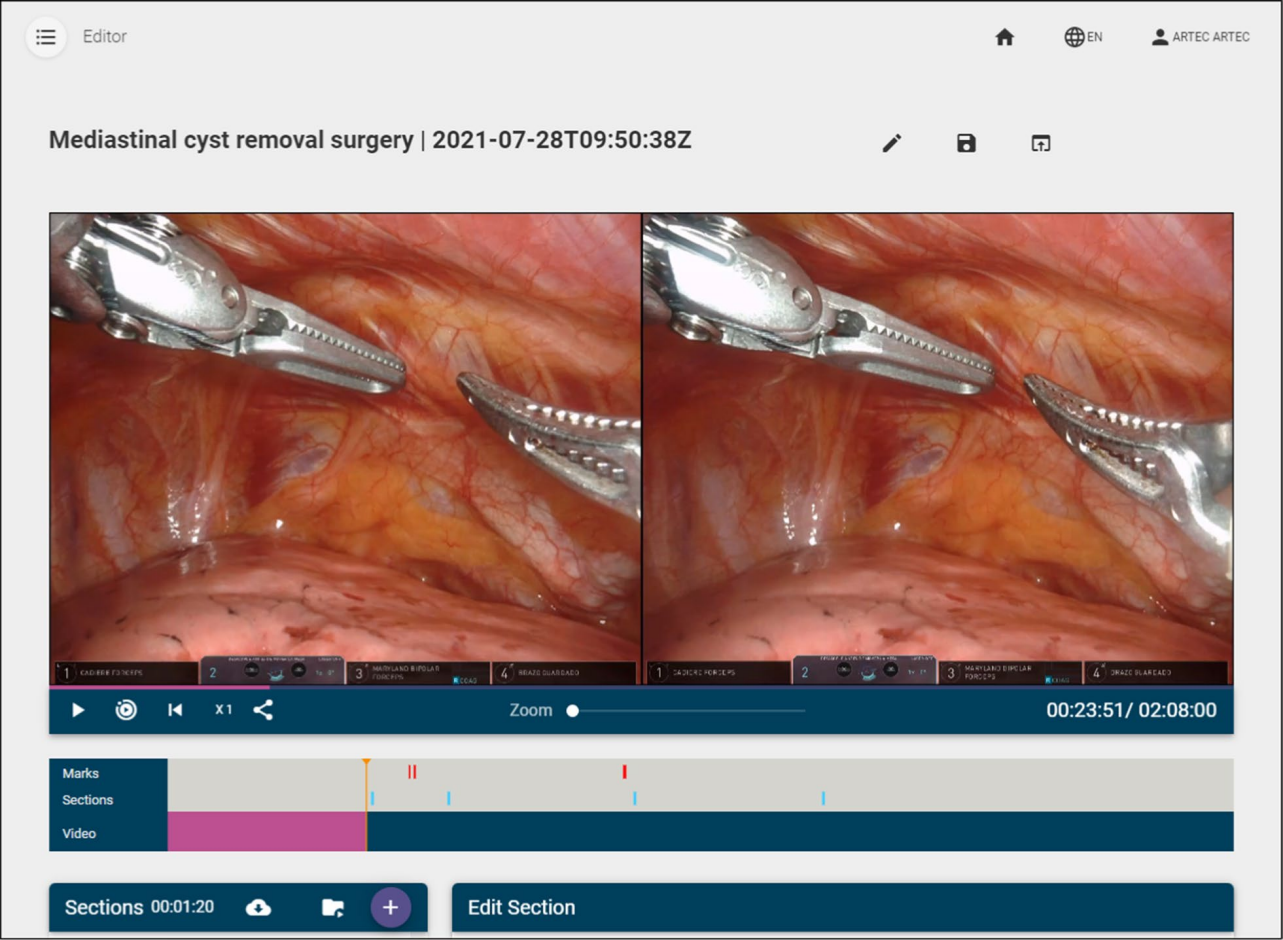
Video editor

Figure 3 shows the video editor. As can be seen, there are three main parts: *annotations* (also called *marks*), *sections* and the video itself, which includes buttons (from left to right) to play/pause the video, to play only the parts of the video marked as sections (and not the whole video), to restart the video, to increase the playback speed and to get a link to download the video. There is also a slider to zoom in/out the video.

The annotation section allows the teacher to highlight a particular element of the video with a virtual bounding box and a text. As the videos do not have depth information, the creation of this virtual annotation needs to be processed in order to calculate the proper horizontal disparity at which the virtual information will be shown in the stereoscopic frame. Otherwise, the annotation will not be perceived at the right depth and the students will not perceive the virtual annotation as part of the stereoscopic video, leading to cybersickness. Thus, the visualization of virtual annotations is carried out by using the Mixed-Reality paradigm, by which virtual elements (in this case virtual annotations) are correctly blended with the real images of the video. The calculation of this stereoscopic disparity is explained here [48].

The idea behind this annotation system is that the user (teacher) chooses an element—an organ, an anatomical element, a surgical tool, etc.—that they want to highlight in order to provide an explanation. This selection is done through a bounding box. Then the user decides how long they want the element to be highlighted for, and the tool automatically tracks the annotated element throughout the video. This way the user does not need to create key-frames for the annotated element, nor do they need to reposition the virtual annotation as the annotated element moves. This annotation tracking process is explained in [48] and uses a state-of-the-art CSRT tracker [49, 50] and an algorithm designed to maintain the horizontal disparity of the virtual annotation at the right values.

**Fig. 4:**
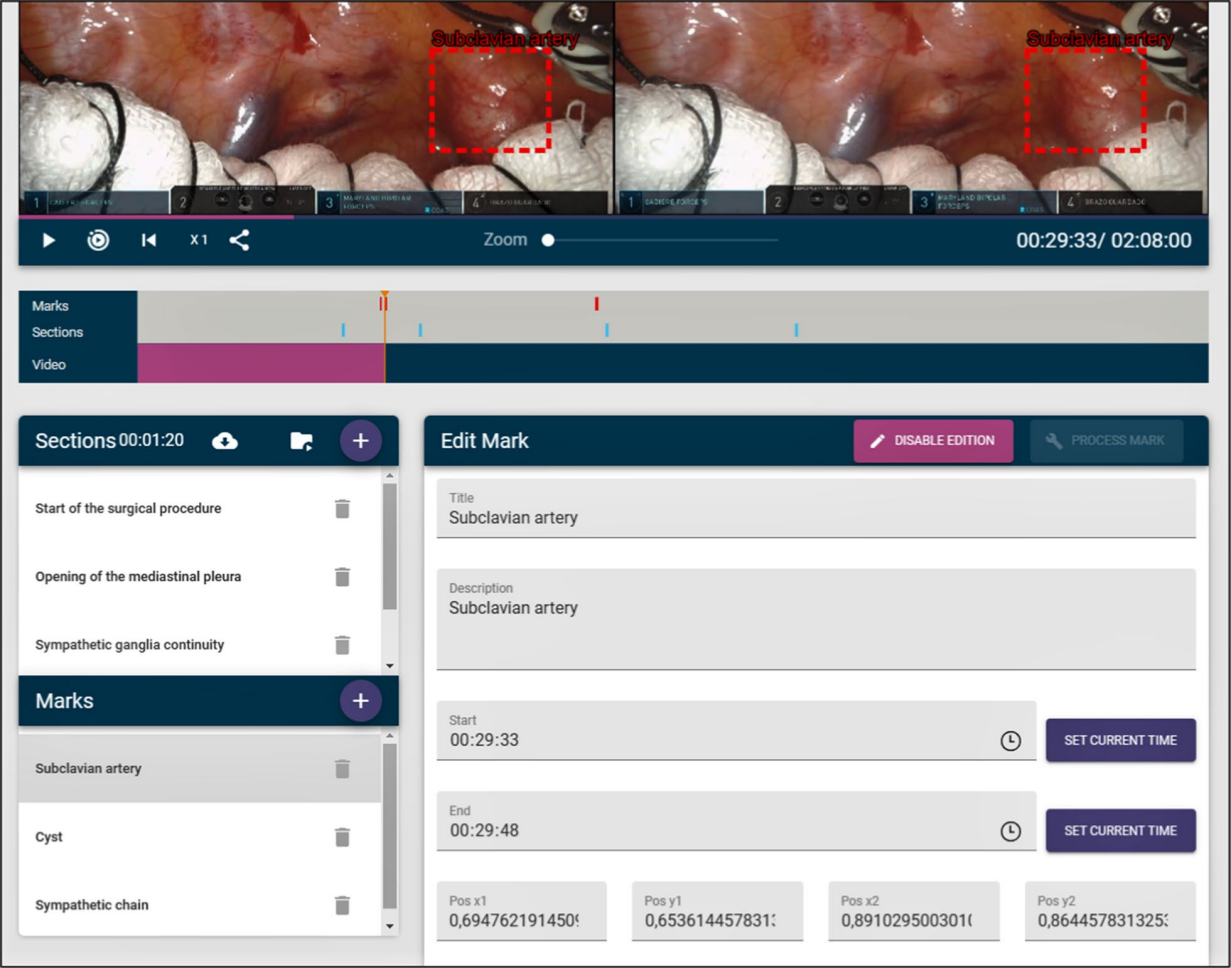
Annotation edition process

**Fig. 5:**
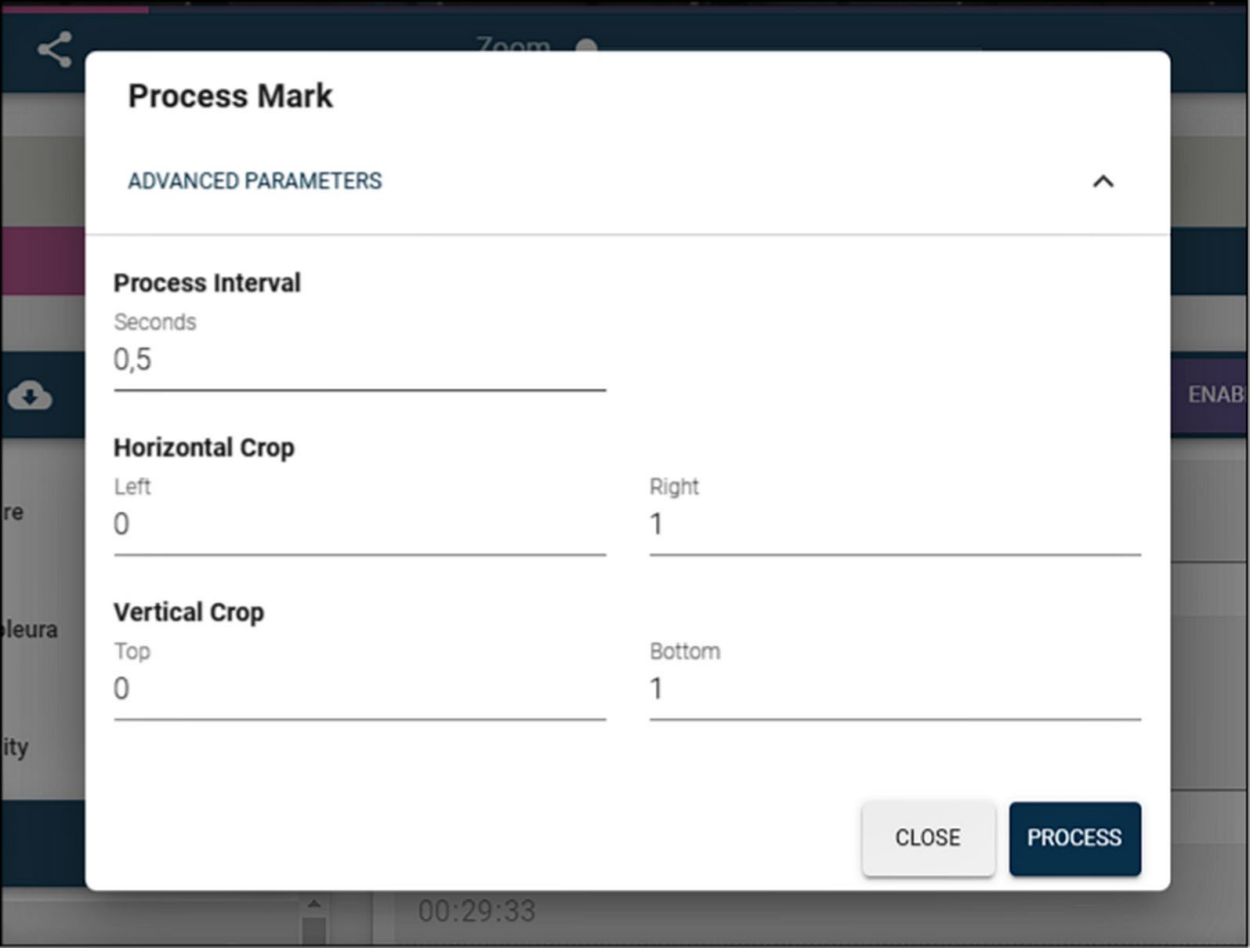
Annotation parameters

It is important to emphasize that this annotation processing is performed asynchronously, because annotation tracking and stereo matching can potentially take some time—especially if the annotation lasts for more than a few seconds—and the application needs to continue working while the annotations are being processed. Figure 4 shows how an annotation can be edited. The user first chooses a bounding box within a selected frame of the video, highlighting some anatomical element or surgical tool. Then, they decide how long—start time and final time—the annotated element should be displayed, providing a text to display and an optional description. Finally, the user commands the application to process the annotation, by clicking the *process annotation* button. This process can be optionally supplied with some parameters, the most important being the update frequency. This update frequency represents how often the tracking system tries to relocate the tracked element. The higher the frequency, the more accurate tracking may be, but the more time it takes to calculate. Values between a tenth of a second and one second are reasonable for this application. The default value is 0.5 s. Figure 5 shows the parameters that can be configured for the processing of an annotation. At the end of the process, the positions and the horizontal disparity of the annotated elements are stored in a database, and thus, this information does not need to be calculated again.

**Fig. 6:**
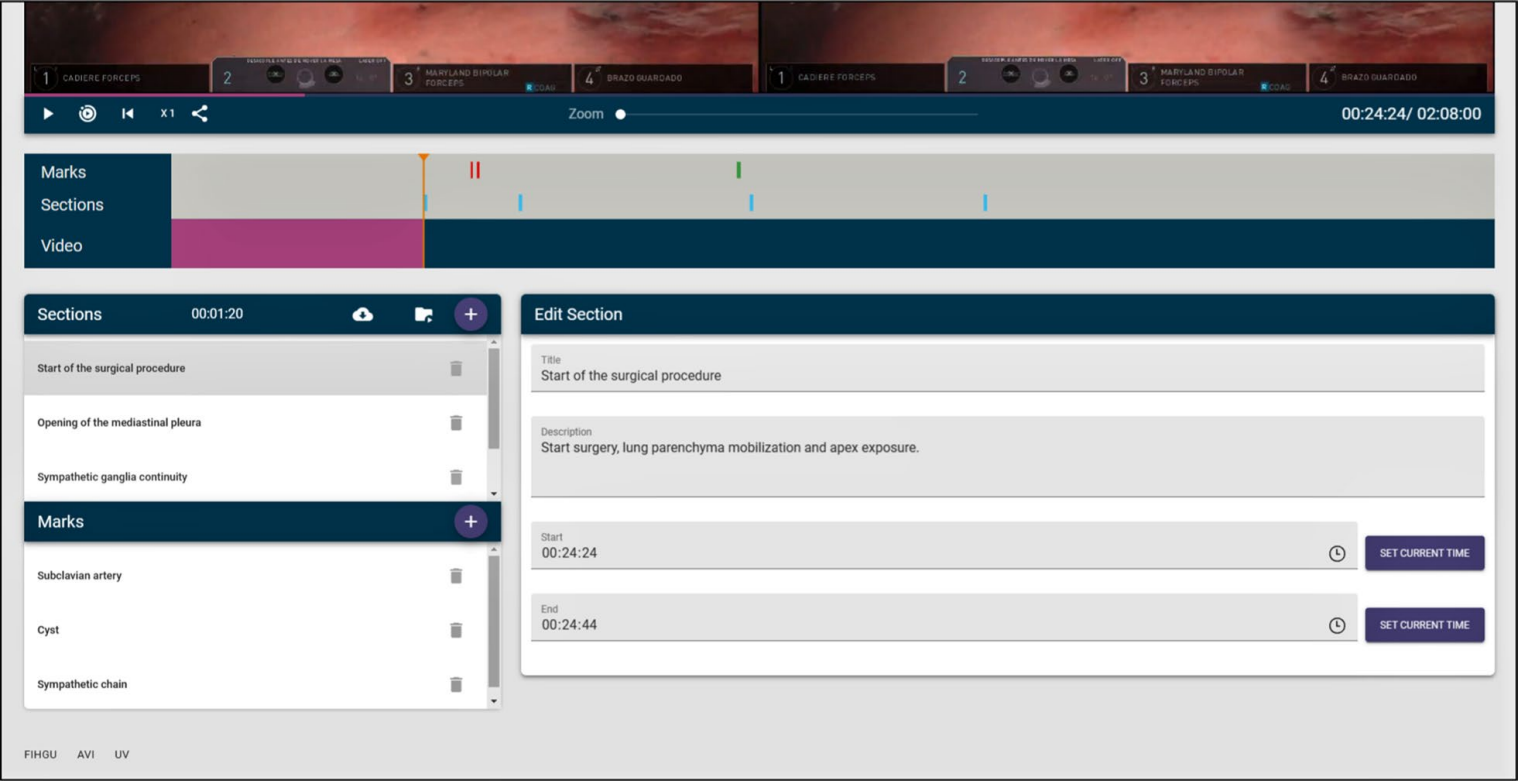
Section creation

On the other hand, the tool allows the teacher to create sections. Many surgical procedures can last for hours and not all the footage has teaching value, since most of the time the surgeon is performing routine work. Thus, it is important to be able to delimit the sections of the video that are of real interest. These sections can have a name and a description and, of course, a start and an end time. More importantly, the teacher or the student can later decide to play the video showing only the parts that belong to a section, skipping the rest of the video. This allows a surgical procedure lasting several hours to be visualized in a few minutes, since only the important parts will be shown. Figure 6 shows how sections can be created.

**Fig. 7:**
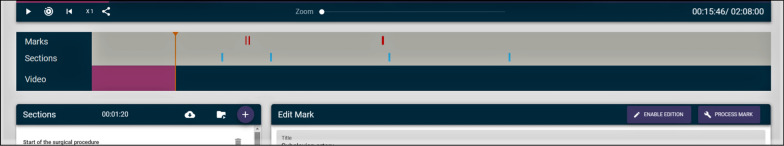
Information bars depicting sections and annotations

**Fig. 8:**
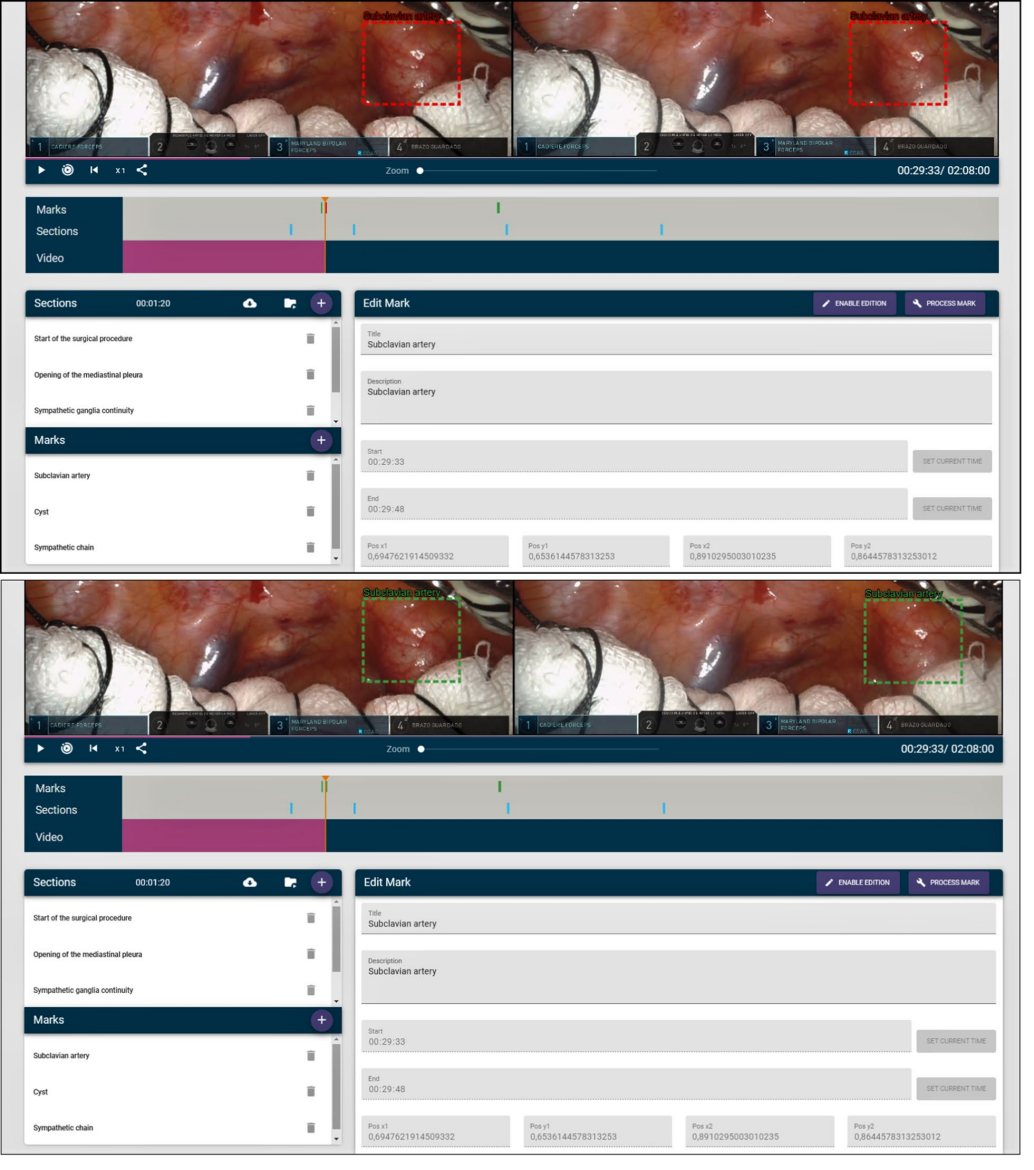
Unprocessed (top) and processed (bottom) annotations. It can be observed that the processed annotation highlights the same area, whereas in the unprocessed annotation there is a mismatch between the left and right bounding boxes

All the information about the annotations and sections of the video is displayed with colored bars. Sections are portrayed in blue, and annotations are displayed in green when they have been processed, in yellow when they are still being processed and in red when they are yet to be processed. Figure 7 shows some of these bars. The colors of the bounding boxes of the annotations also follow this convention, so that the teacher can know when an annotation has been previously processed, is being processed or remains to be processed. Annotations that have not been processed are shown in the editor, but no tracking or calculation of the horizontal disparity is performed. Therefore, they will not be shown to the students if the video is broadcast to them. Only processed annotations will be included. Figure 8 shows both a processed and unprocessed annotation.

Finally, as discussed at the beginning of this section, there are a series of buttons to play/pause the video, to play it at different speeds (1x, 2x, 4x, 8x), to play only the parts of the video belonging to a section and also a button to zoom in–out in case the stereoscopic video is incorrectly aligned. There is also a button that generates a link, so that the video can be shared with a single URL and downloaded—although an authorized user is needed to be able to use this link. Figure 3 shows all these controls. Although the editing features are, of course, not available for students, the buttons shown in Fig. 3 are available for both students and teachers, since both roles are granted permission to play all published videos, and the viewer tool shares most of these buttons, as will be shown in "Stereoscopic RAS viewer" section.

### Stereoscopic RAS viewer

Once the video has been edited by the teacher and enhanced with virtual content, it can be moved from draft status to published status. Only published videos can be shared and broadcast to the students, although published videos can still be further edited. Once a video is published, a display button appears next to it, allowing the teacher to create a virtual room to host the collaborative display of the video to the students.

**Fig. 9:**
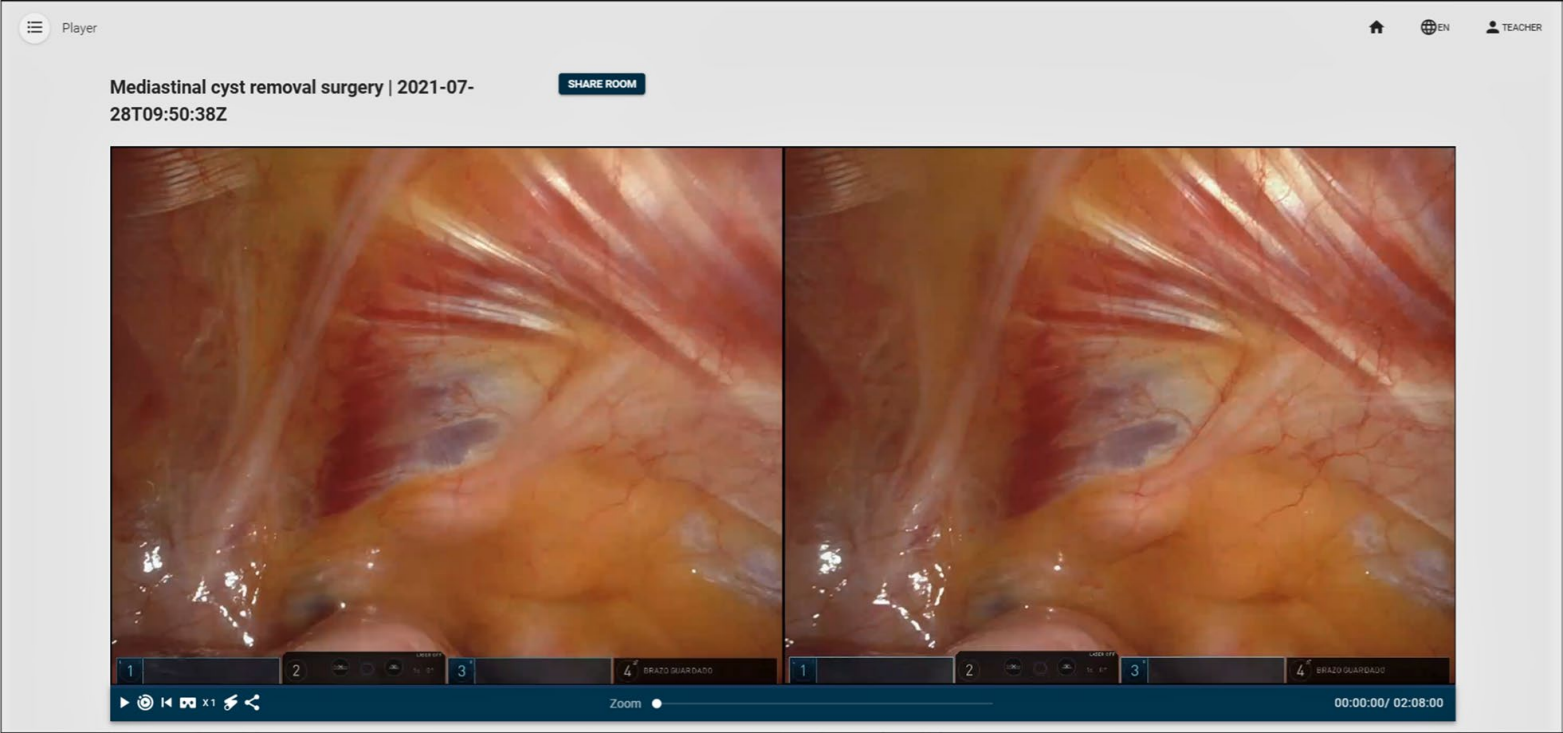
Stereoscopic RAS viewer from the teacher’s perspective

**Fig. 10:**
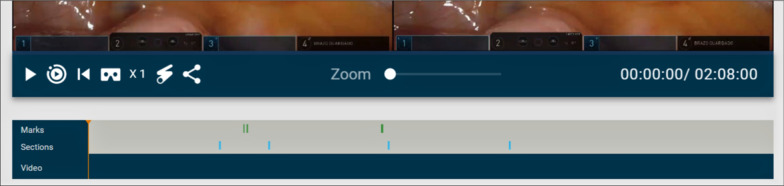
Close-up of Fig. 9 showcasing the control buttons

**Fig. 11:**
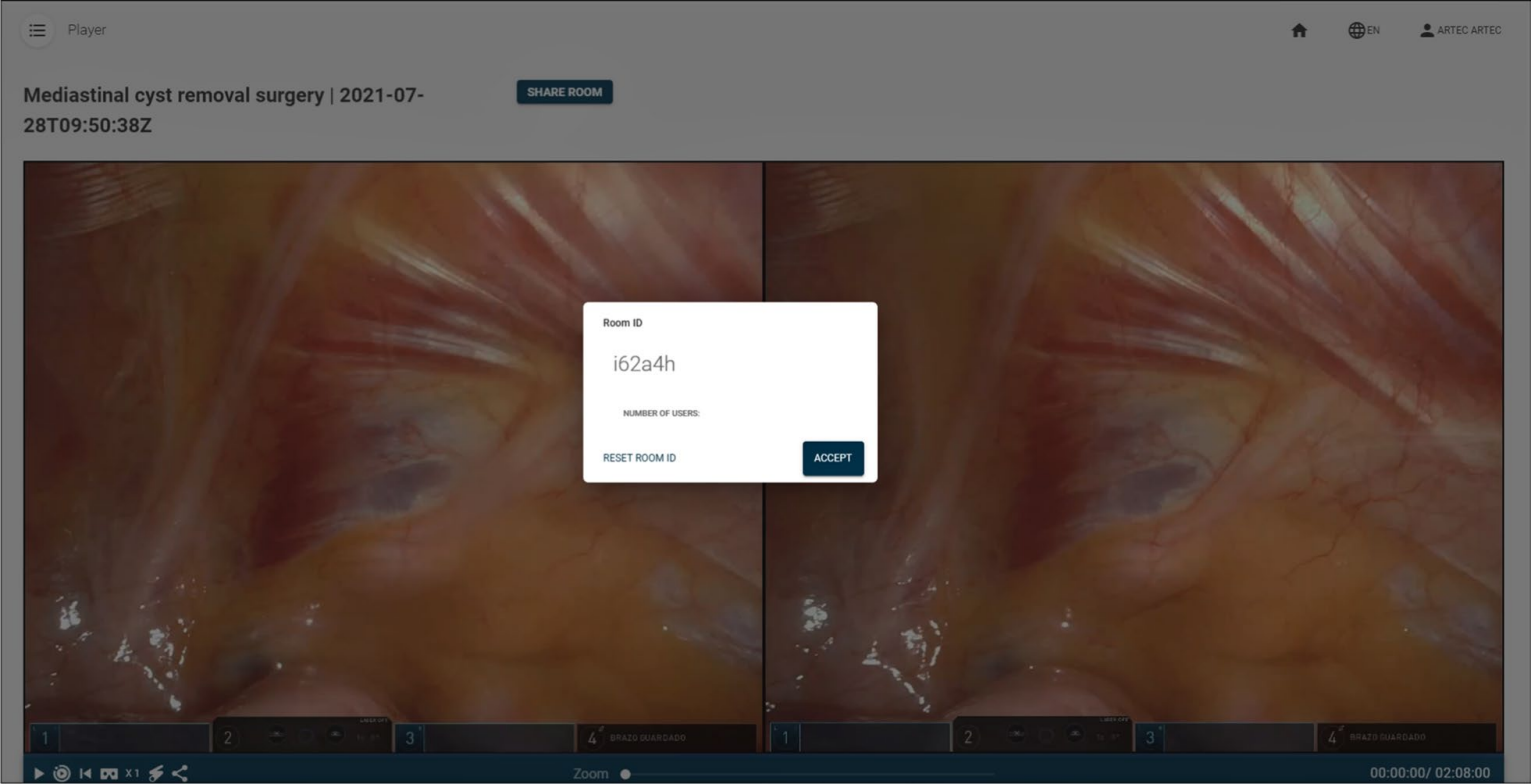
Room creation by the teacher

The view from the teacher’s perspective is very similar to the video editor. The only difference is that neither annotations nor sections can be modified. They can only be consulted and watched. The video controls (play, pause, change video speed, play sections, generate link) are the same as in the editor’s view. However, there are now two additions: one button to share a pointer and another button to display the video in VR mode (see Figs. 9, 10). The pointer button introduces a red pointer onto the screen and allows the teacher to share this pointer with the students. This way, the teacher can use it to highlight certain parts of the video. The VR mode button allows the teacher to visualize the video stereoscopically. This mode can be used to watch the video with a high-end HMD display, such as an HTC Vive, but it can also be used to watch the video using a smartphone or a tablet, whilst wearing Cardboard VR glasses. The target display is automatically detected. Finally, there is a button to generate a room code and broadcast the MR-enhanced video to the students. Figure 9 shows the RAS viewer from the teachers’ perspective. Figure 11 shows how the teacher creates a collaborative room so that the students can access the room.

**Fig. 12:**
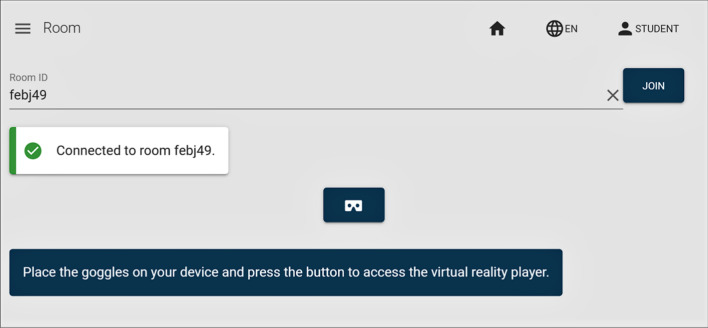
Room access from the student’s perspective

The process of sharing a collaborative display is very simple. First the teacher decides to share a video by creating a virtual room with a corresponding room code. Then, the teacher communicates this code to the students. This should be done outside the tool (verbally or by other means). Finally, students enter the room and start visualizing the video. To visualize it in stereoscopic mode, they need to click on the glasses icon (see Fig. 12). If they are using a smartphone, which is the expected use case, they will be able to watch the video stereoscopically using a simple pair of Cardboard VR glasses. In any case, the reproduction of the video (play, pause, speed, etc.) is always controlled by the teacher. Figure 12 shows the access to the virtual room from the students’ perspective.

**Fig. 13:**
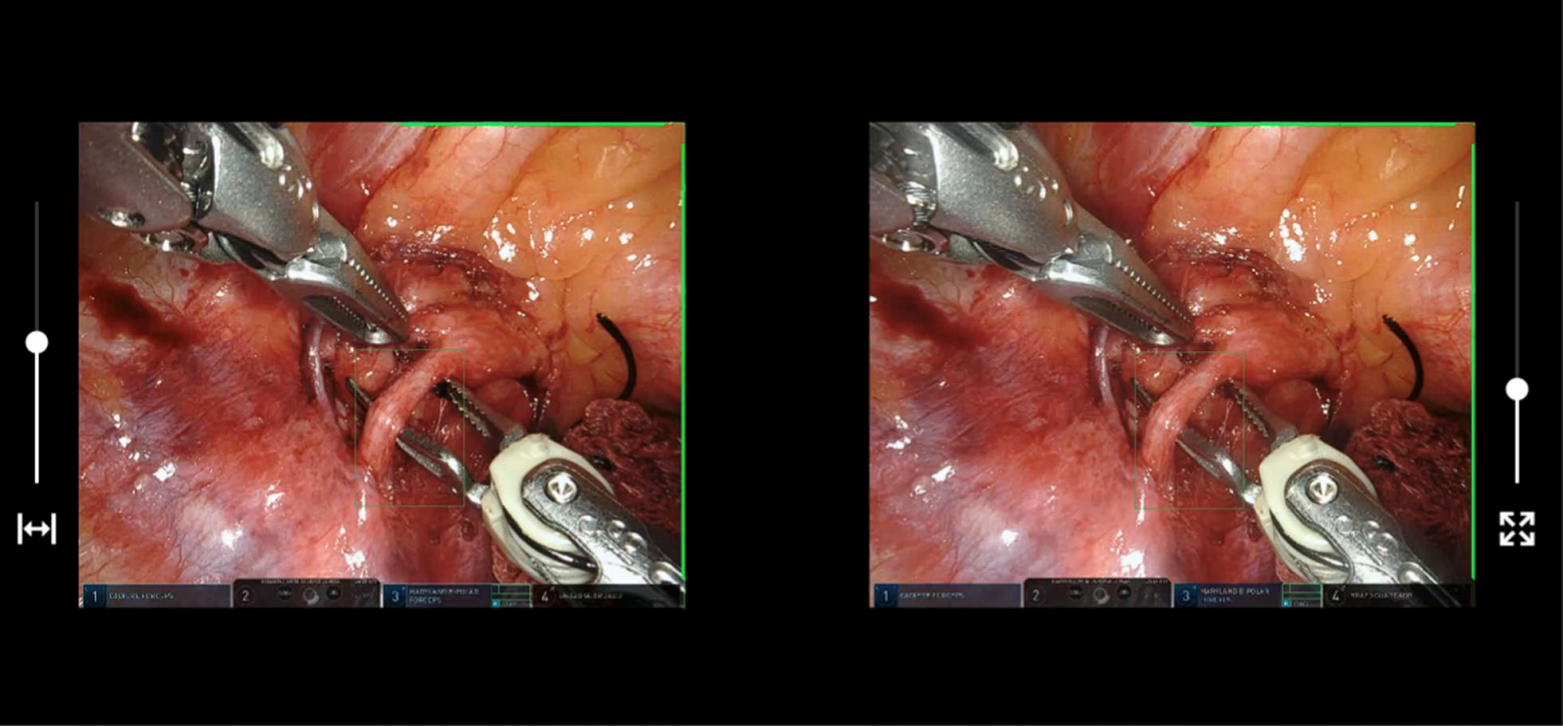
MR mode with the disparity (left slider) and zoom (right slider) controls

Since there are many different smartphones with a plethora of display sizes, the MR viewer that our tool provides allows the adjustment of the horizontal disparity and the zoom of the video so that a comfortable view is achieved (see Fig. 13). Thus, it is possible to adjust the image to fit almost any mobile device, any type of VR glasses and any user.

In collaborative mode, the teacher will always be in control of the reproduction. Whatever control (play, pause, play sections, etc.) they use will be translated into the students’ view. This way all the students share the same view, and the teacher can be sure that what he/she sees is what the students can see.

It is important to emphasize that the students and the teacher do not need to share the same physical location. They do not even need to share a common computer network, since the only requirement is that they can establish an IP-based communication. However, it makes much more sense for them to share a common physical room. This way, the teacher can easily combine verbal instructions with visual information.

### Students’ view

The students enter the application in the same way teachers do, albeit their account does not have upload or edit permissions. Students have two major options, to watch previously uploaded videos (without any collaborative feature) or to join a room class for a collaborative display. In the former, the interface is almost the same as the one previously described for the teacher, but without the editing options. Students can see a list of available videos including a search mechanism by: organs, techniques, etc. Once a video is selected, the student can watch the video with all the related information, including sections and annotations. The reproduction options are similar to the ones previously described, so a student can watch, i.e., a specific section, an annotation or the summary of the video where only sections are played. This visualization interface also includes the stereoscopic visualization mode, so any student can use his/her own mobile device to watch the desired content in an immersive manner. In the latter case they can connect to the shared view where a teacher will control the visualization. Once the teacher casts a surgical procedure and creates a room, students can access it by typing a room code. This room code is always created by the teacher—in fact it is generated automatically by the application at the request of the teacher—and gives access to whatever content the teacher chooses to display.

### Computer architecture

**Fig. 14:**
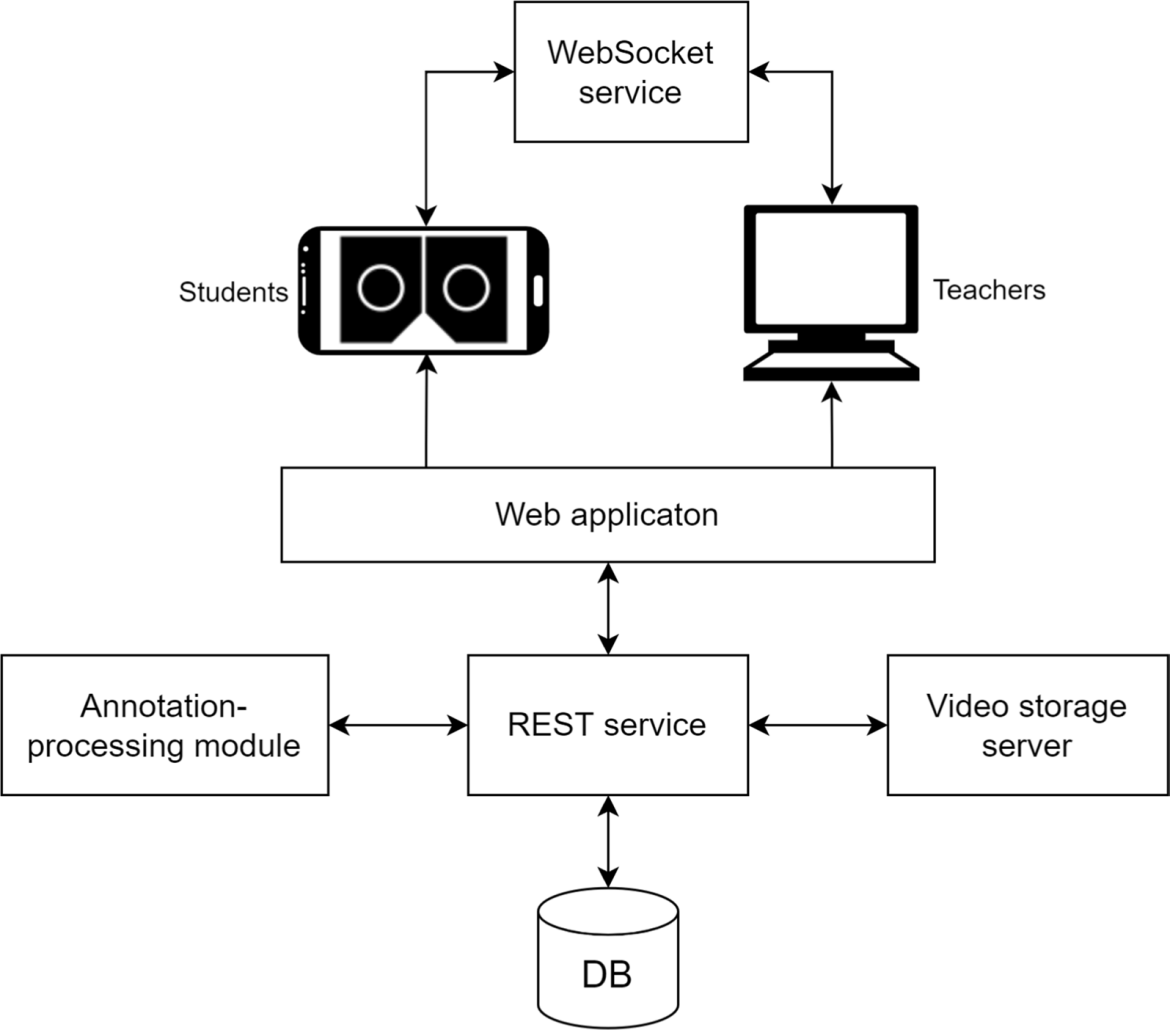
Computer architecture of the proposed MR-based RAS training application

Our RAS training tool needs a computer infrastructure to work. The system is composed of six main modules: a web application, a REST service, an annotation-processing module, a MySQL database, a video storage server and a WebSocket service. Figure 14 shows the computer architecture of the proposed system.

The web application provides the interface to the users. This is an HTML5-based application developed with Vue.js, a JavaScript-based framework for web application development. This web application serves as a front-end for the system, where the users perform all the actions. It is composed of several modules that provide the functionalities for login, intervention uploading, editing and visualization. For stereoscopic video visualization the Three.JS library is used. Therefore, WebGL support is needed on the browser’s side. In order to provide a full screen experience, and avoid disturbing UI elements, the Fullscreen API is also used.

**Fig. 15:**
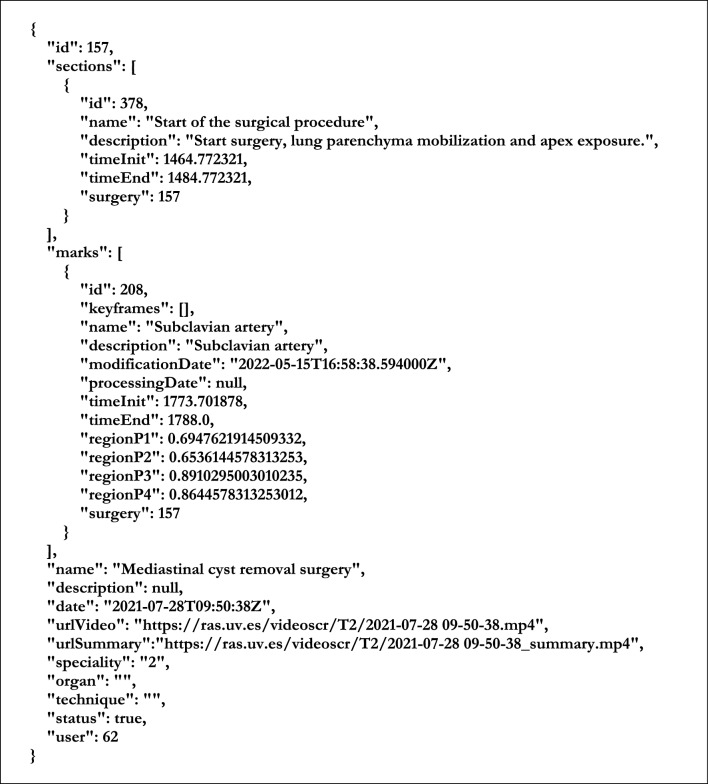
Example of a JSON file describing a surgical procedure, including one (unprocessed) annotation and one section definition

The REST service was developed with the Python-based Django framework. It exposes a public API, through which the web application and the rest of the modules can communicate with the database by means of HTTP requests. These requests, and the corresponding responses, use JSON format. The REST service also manages the user authentication with expiring JWT tokens. Figure 15 shows the JSON-based format used in our application to communicate information about the surgical intervention between modules.

The REST service module communicates with the annotation-processing module whenever an annotation is created and requested to be processed. This module is an OpenCV-based procedure written in Python that calculates all the positions of the annotations asynchronously, so that the rest of the modules of the system can continue working regardless the completion status of this annotation process. The results of this process (the key frames of the highlighted elements with their bounding boxes and annotation texts) are sent to the REST service to be stored in the database.

The WebSocket service provides real-time communication between users of the web application. This service manages the different virtual rooms and broadcasts messages from the teacher to the students. This real-time communication allows the teacher to control the video playback on the students’ devices. It also communicates the position of the shared pointer. This module is separated from the web application module and uses Node.js and Socket.IO to manage the communication.

**Fig. 16:**
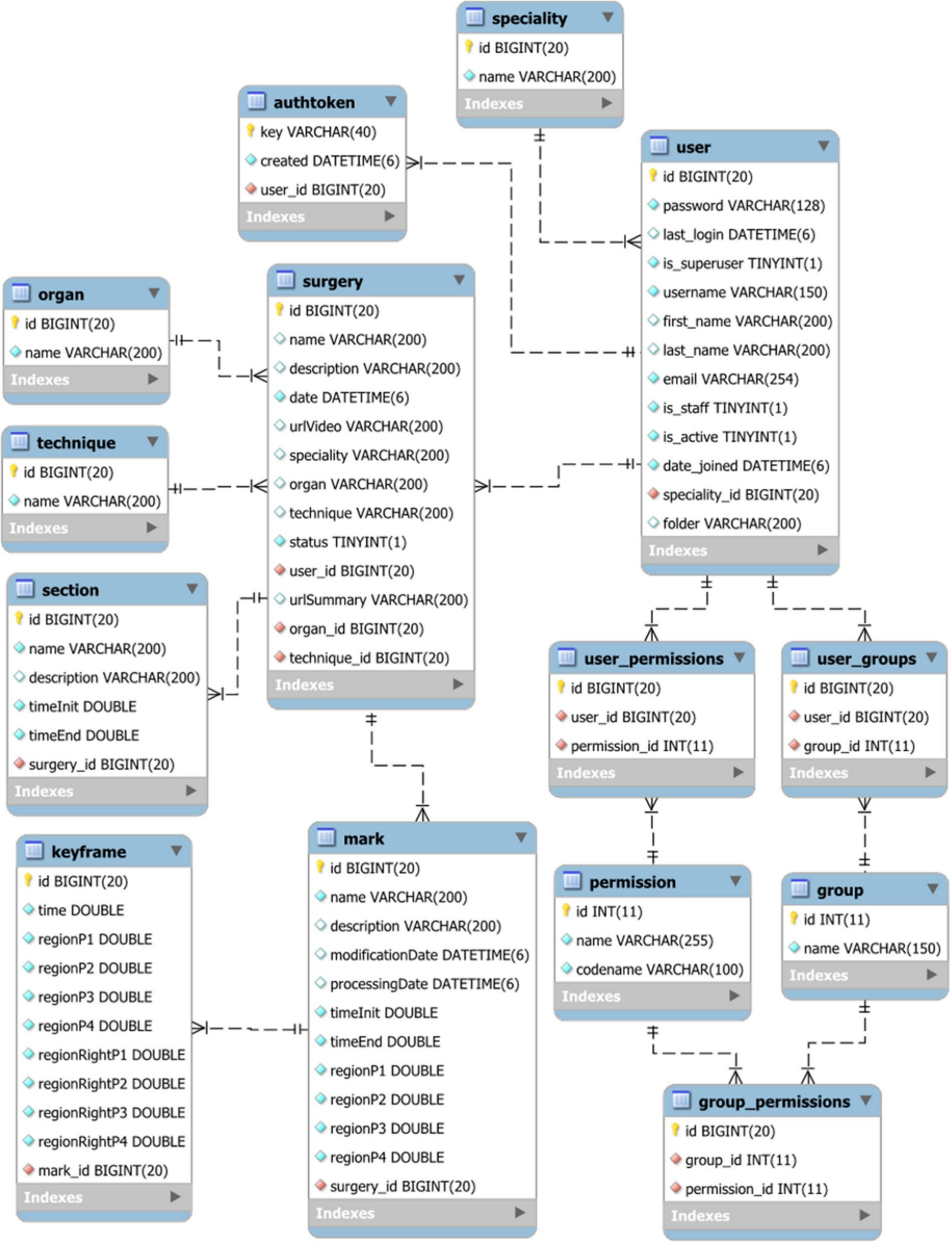
Database diagram

The database stores all the information about the users and the surgical interventions, so each surgeon can have a customized view of the application. Figure 16 shows the database diagram of the application. As can be seen, there are tables for storing information about surgical interventions (tables *surgery*, *organ*, *technique*, *specialty*), users and authentication management (tables *user*, *user_permissions*, *user_groups*, *permission*, *group*, *group_permissions*, *authtoken*), video sections (table *section*) and video annotations (tables *mark* and *keyframe*).

Although the database does store all the information about the videos, it does not store the videos themselves. Instead, it links to the video storage server where all the stereoscopic videos are saved. This server is protected with encrypted access since these videos are sensitive health data of real patients. All of them need to be stereoscopic FullHD videos, in side-by-side format. Thus, the video server stores 3840 × 1080 videos. The acquisition of the footage from the surgical robot is beyond the scope of this work, but our tool is designed to work with any surgical hardware.

### Mixed-reality infrastructure

As aforementioned, the tool is a web-based application. It allows access using computers, tablets and mobile phones. The only requisite is to have a web browser compatible with WebGL (something that all recent web browsers meet). In fact, the expected scenario is that teachers use a desktop computer to enter the application, edit the videos, add virtual information to them and broadcast the MR-based teaching assets, while the students use their mobile phones to watch the training materials created by the teachers. This is possible because of the use of the WebSocket-based communication system by which, any person with an IP-capable device and a compatible web browser can connect to the teacher’s room.

**Fig. 17:**
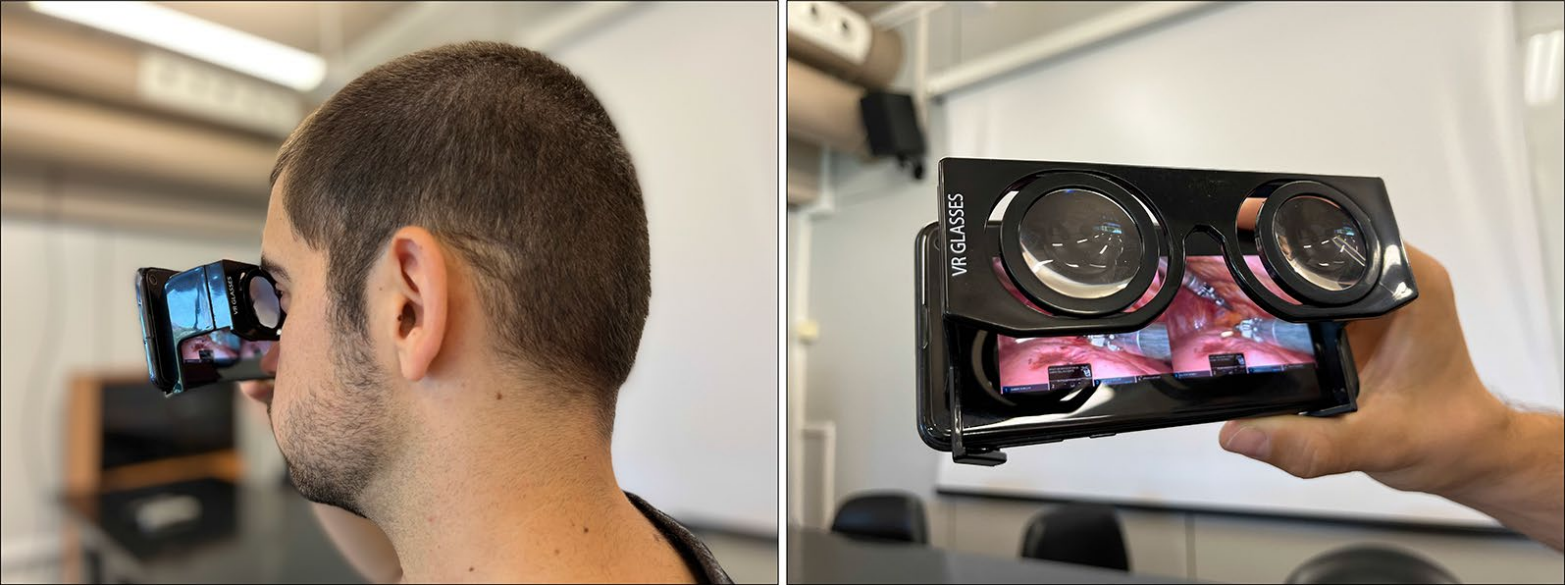
Snapshot of a student using the application (left) and close-up view of the mobile-based viewer using a pair of Morgan VR glasses and an Android smartphone (right)

This is ideal for the teachers, as they do not need a dedicated computer—with specific software—to run the application. However, the key advantage of this web-based infrastructure is that it allows students to view the training videos edited with this tool on almost any device, including their own mobile phones. This is of the utmost importance, since most professional MR-based medical tools are developed for expensive devices, such as Head-Mounted Displays or stereoscopic displays. Our tool could be used with a simple smartphone. All the students need is a mobile phone with a web-browser and Cardboard VR glasses. These VR glasses can be bought for less than one dollar. Figure 17 shows a student using our application on a smartphone wearing VR glasses. Our tool works both in Android and iOS smartphones.

## Experimental study

Once the tool was ready for deployment, a quantitative subjective evaluation with real users was performed. To this end we deployed all the back-end modules of our application in one of the servers in our university and arranged a test session at the Hospital General de Valencia, where a simple laptop computer was used to display the web application. This hospital is one of the oldest hospitals in Spain, and it is one of the reference centers in robotic surgery in Spain. The experiments took place as part of a master's session in robotic surgery, since this hospital offers a Master’s Degree in robotic surgery. The students on this master’s course are surgeons who want to be trained in robotic surgery. Therefore, it is the perfect place and population to test our application. Fifteen people participated in the experiment, which was completely voluntary. There was no financial compensation for participation and the experiment lasted for one hour.

For the experiment, a teacher, who is an expert in RAS, used our tool to show a surgical intervention. Previously, this person had uploaded the video of the surgical procedure and had edited it, so that the most important parts of the surgery were explained to the students. The intervention was a robotic resection of a mediastinal cyst in the thoracic outlet, in the specialty of Thoracic Surgery, performed with a Da Vinci surgical robot.

Four sections were created in this video:

1. *Start of the surgical procedure* indicates the beginning of the surgery, after port placement and docking. You can see the mobilization of the lung parenchyma to achieve proper exposure of the lesion at the apex.
2. *Opening of the mediastinal pleura* once the cyst and the surrounding anatomical structures have been identified, the mediastinal pleura is incised over it to begin the resection.
3. *Continuity with the sympathetic chain* it can be seen how the cyst seems to depend on the sympathetic chain. This indicates that the only way to perform a complete resection of the cyst would be to section the sympathetic chain at that level.
4. *Rib release* adhesions from the lower pole of the cyst to the rib are released.

Three annotations were also created: these three marks indicate anatomical structures that are particularly important in this surgery.

1. *Subclavian artery* this is a large caliber artery. Its injury can cause massive bleeding and be a life-threatening emergency.
2. *Cyst* this is the target lesion.
3. *Sympathetic chain* At the level of T1 (where the cyst is located), injury to the sympathetic chain, or the stellate ganglion, should be avoided due to its secondary effects such as Horner´s Syndrome (ptosis, miosis, anhidrosis).

**Fig. 18:**
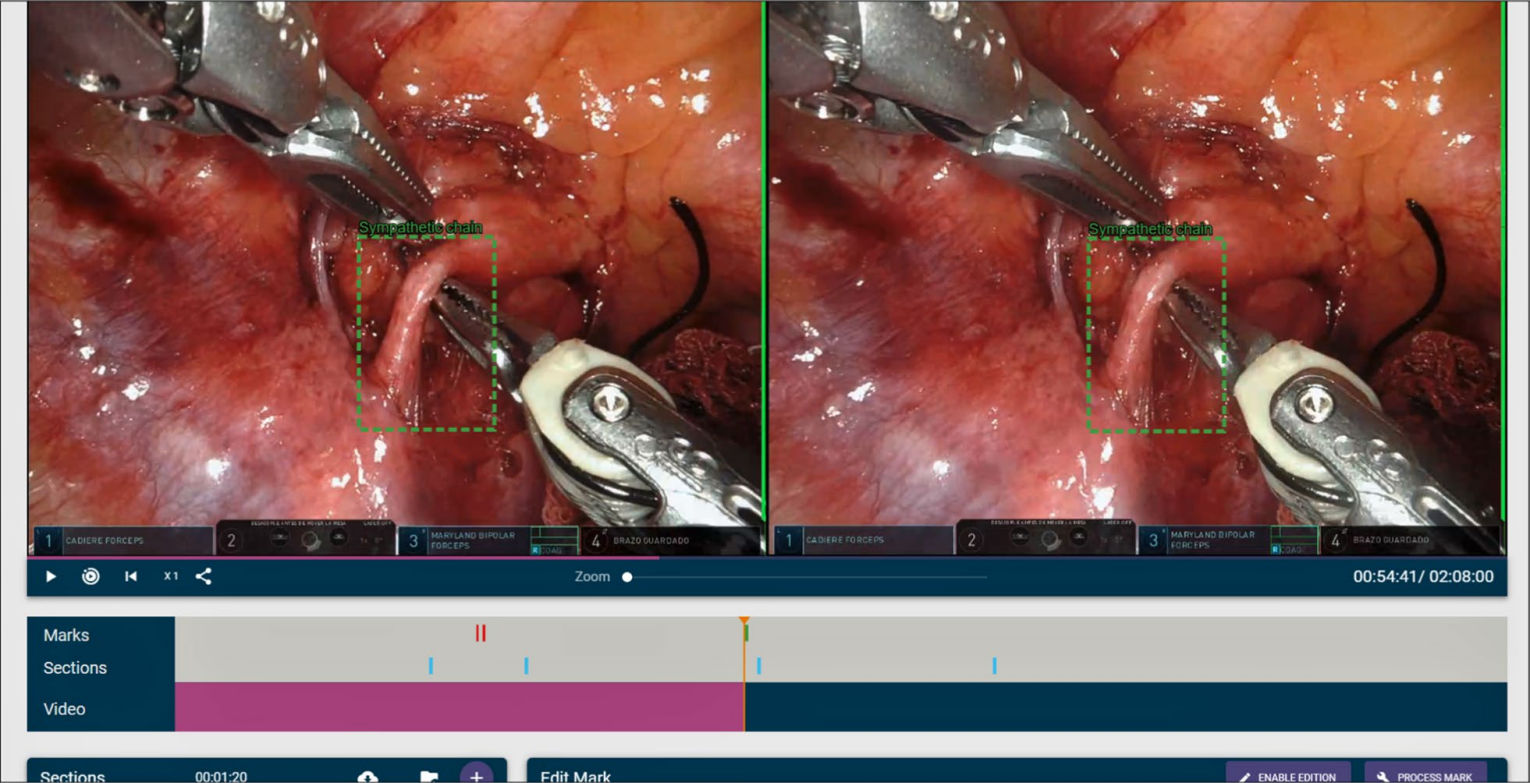
Snapshot of the video used during the experiment

Figure 18 shows a snapshot of the video with one of the annotations.

**Fig. 19:**
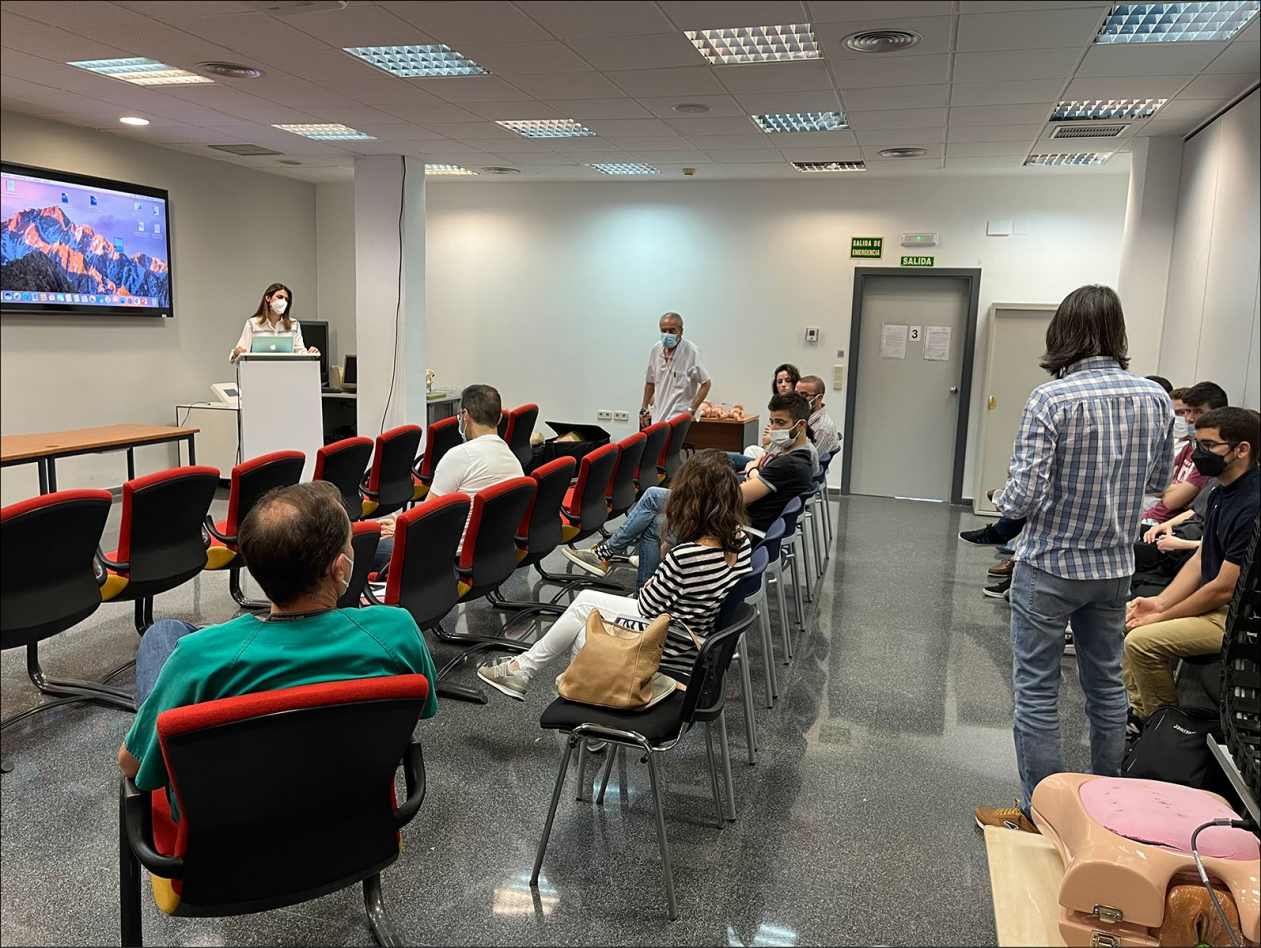
A picture of the test at Hospital General de Valencia

After a brief introduction to the tool, the teacher created a virtual room to show the previously MR-enhanced video. Then, we handed out smartphones to the students so they could use them to connect to the room. They tested the application as they followed the teacher’s explanations. Figure 19 shows a picture of the test. At the end of the video class, the students were asked to answer a questionnaire containing 15 questions. Table 1 shows this questionnaire. The first 14 questions were 7-option Likert questions, in which the anchors were: *strongly disagree* (1), *disagree* (2), *somewhat disagree* (3), *neutral* (4), *somewhat agree* (5), *agree* (6) and *strongly agree* (7). These questions try to measure if the participants feel that the elements of the tool are useful as training assets. Thus, some questions address the use of annotations, some deal with the sections, some with the pointer, and some are general questions about the usefulness of the tool as a RAS training device. The last question is a 0–10 question to rate overall experience with the tool.

**Table 1:** Questionnaire

Question #	Question text
1	With this type of video, I could get a better understanding of how surgical robots should be used to perform operations
2	The depth perception of the videos was correct
3	The depth perception of the videos helps to better understand the position and orientation of the surgical material in relation to the patient's anatomical elements
4	My perception of depth regarding the annotations added to the videos was correct
5	The annotation system provided by the tool can be useful to highlight specific aspects of the surgery
6	I consider that the annotations created correctly highlighted the annotated element and moved with it adequately
7	I felt that the annotations were well integrated and well visualized within the video
8	I believe that the use of sections within a video helps to visualize the video more quickly
9	The tool's pointer helps to point out elements of interest in the video
10	The perception of the pointer was correct
11	I believe that the annotations that can be added to the videos are useful for training purposes
12	I believe that the use of sections to highlight certain parts of the surgery is useful for training purposes
13	The use of 3D videos for training purposes in the field of robotic surgery is an important improvement over the use of 2D videos
14	With this tool, the training of surgeons in the field of robotic surgery can be improved in a simple, fast way
15	Rate, from 0 to 10, the usefulness of this tool

Since the evaluation was conducted with students, they only assessed the resulting video and the use the teacher made of the application. Therefore, this evaluation does not assess the authoring part of the application, as this part is only used by the teachers.

The results of these questions were processed with SPSS 26 software in order to extract conclusions about the responses of the participants. First, we conducted normality tests, in order to decide if we could use parametric tests or not. Then, we applied one sample parametric and non-parametric tests to compare the results against a reference value. 0.05 was used as a threshold for statistical significance.

## Results and discussion

The first meaningful result is that the evaluation session was conducted without incident; every user was able to use the stereoscopic visualization and all the comments during the session were very positive, highlighting the novelty of the visualization system. There were no noticeable issues related to cybersickness or uncomfortable feelings while using the devices.

**Fig. 20:**
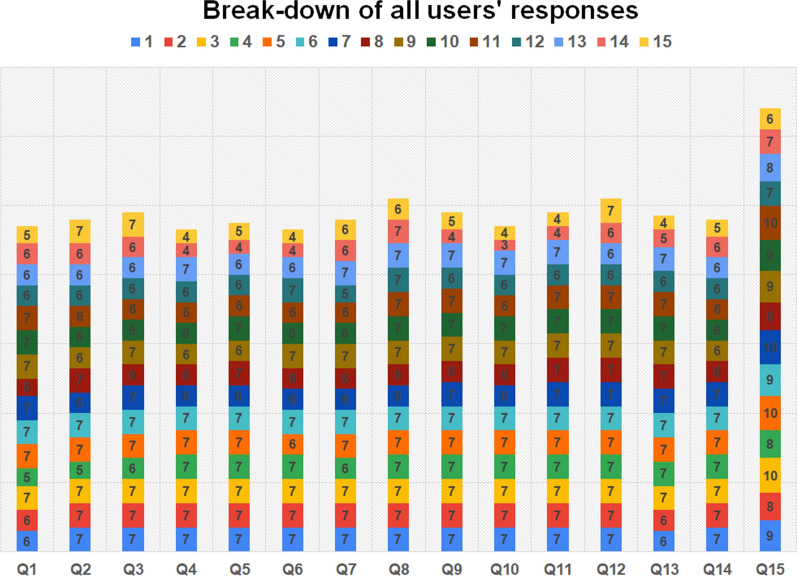
Break-down of all user responses in bar plots

Regarding the quantitative analysis, Table 2 summarizes the results of the 15 questions. As can be seen, all 7-option Likert questions provide average results above 6 with small standard deviations (in most cases less than 1). No value lower than 4 was obtained for any of the questions by any participant. A similar situation occurred for question #15. Figure 20 shows a detailed break-down of all user results.

**Table 2:** Summarized results with some measurements of central tendency

Question #	Average	Standard deviation	Minimum	Maximum	Median	Interquartile range
1	6.267	0.799	5	7	6	6–7
2	6.400	0.632	5	7	6	6–7
3	6.533	0.516	6	7	7	6–7
4	6.200	1.014	4	7	6	6–7
5	6.333	0.900	4	7	7	6–7
6	6.200	1.014	4	7	6	6–7
7	6.400	0.632	5	7	6	6–7
8	6.800	0.414	6	7	7	7–7
9	6.533	0.915	4	7	7	6–7
10	6.267	1.223	3	7	7	6–7
11	6.533	1.060	4	7	7	7–7
12	6.800	0.414	6	7	7	7–7
13	6.467	0.915	4	7	7	6–7
14	6.400	0.632	5	7	6	6–7
15	8.533	1.246	6	10	9	8–10

Although the results are clearly positive, in order to obtain a more detailed picture of the opinions of the participants, we first applied normality tests to the data, so that we could decide if parametric or non-parametric tests should be used to analyze the data. Table 3 details these normality tests. We performed both Kolmogorov–Smirnov and Shapiro–Wilk tests. The results of both tests are consistent with each other. Only for the results from question #15 can the null hypotheses (there is no significant difference between the variable and a normal distribution) be accepted and the variable can be assumed to follow a normal distribution. In the rest of cases (questions 1–14) the null hypothesis cannot be accepted and normality cannot be assumed. Thus, parametric tests could be used only for question #15.

**Table 3:** Normality tests

Question #	Kolmogorov–Smirnov		Shapiro–Wilk		Result
	Statistic (D)	Significance	Statistic (W)	Significance	
1	.287	.002	.783	.002	Non-normal
2	.295	.001	.761	.001	Non-normal
3	.350	.000	.643	.000	Non-normal
4	.289	.002	.730	.001	Non-normal
5	.304	.001	.748	.001	Non-normal
6	.289	.002	.730	.001	Non-normal
7	.295	.001	.761	.001	Non-normal
8	.485	.000	.499	.000	Non-normal
9	.428	.000	.596	.000	Non-normal
10	.326	.000	.659	.000	Non-normal
11	.470	.000	.494	.000	Non-normal
12	.485	.000	.499	.000	Non-normal
13	.387	.000	.658	.000	Non-normal
14	.295	.001	.761	.001	Non-normal
15	.179	**.200**	.908	**.126**	Normal

Bold values highlight significant figures

In order to check how favorable the opinions of the participants were, we performed a one sample Wilcoxon signed-rank test for questions 1–14. This non-parametric test allows us to know if the answers to each of the questions are significantly above a certain threshold. Three thresholds were used: 4, 5 and 6. Table 4 shows the results of these tests. All the questions present a median answer significantly above *neutral* (4). All of them also have a median result significantly above *somewhat agree* (5), and half of them (questions #2, #3, #7, #8, #9, #12 and #14) show a median result that is significantly above *agree* (6). These questions deal with depth perception (question #2), with the usefulness of 3D visualization to understand the position and orientation of the surgical material in relation to the patient's anatomical elements (question #3), with the integration of the annotations within the video (question #7), with the usefulness of the use of sections (questions #8 and #12) and the pointer (question #9), and the usefulness of the tool as a training asset (question #14). These are very good results that confirm that the surgeons consider this tool to be a very useful one. The questions that show some room for improvement were question #4 (depth perception about annotations), question #5 (usefulness of the annotations), question #6 (annotation tracking), question #10 (depth perception about pointer), question #11 (usefulness of the annotations for training) and question #13 (difference between 2 and 3D). Question #4 and question #6 deal with how to track and show the annotations. It is quite complicated to track internal body elements, since object trackers are not designed with this use case in mind. It is also hard to calculate the exact horizontal disparity of the annotation with endoscopic cameras that are constantly moving. Therefore, some users may notice some discomfort when occlusions or fast movements hinder the calculation of this annotation process. In any case, both display an average of 6.2, which is quite high.

**Table 4:** One sample Wilcoxon signed-rank test non-parametric tests for Questions 1–14

Question #	Test value = 4		Test value = 5		Test value = 6	
	Statistic (Z)	Significance	Statistic (Z)	Significance	Statistic (Z)	Significance
1	3.464	**.001**	3.153	**.002**	1.265	.206
2	3.487	** < 10**^**–3**^	3.391	**.001**	2.121	**.034**
3	3.508	** < 10**^**–3**^	3.508	**< 10**^**–3**^	2.828	**.005**
4	3.272	**.001**	2.982	**.003**	.687	.492
5	3.384	**.001**	3.170	**.002**	1.387	.166
6	3.272	**.001**	2.982	**.003**	.687	.492
7	3.487	**< 10**^**–3**^	3.391	**.001**	2.121	**.034**
8	3.626	**< 10**^**–3**^	3.626	**< 10**^**–3**^	3.464	**.001**
9	3.491	**< 10**^**–3**^	3.361	**.001**	2.000	**.046**
10	3.342	**.001**	2.745	**.006**	1.136	.256
11	3.500	**< 10**^**–3**^	3.385	**.001**	1.728	.084
12	3.626	** < 10**^**–3**^	3.626	**< 10**^**–3**^	3.464	**.001**
13	3.442	**.001**	3.284	**.001**	1.807	.071
14	3.487	** < 10**^**–3**^	3.391	**.001**	2.121	**.034**

Bold values highlight significant figures

None of the questions received a value below 6, and some of them received average values close to the maximum possible value (7). Therefore, we can affirm that the evaluation of the tool is satisfactory.

Finally, we analyzed question 15. Given that the average value for question #15 is 8.533, it is quite obvious that this value is significantly higher than 5. In any case, we conducted a series of t-tests in order to find out how high a test value could we reach whilst still getting significant results. Table 5 shows these t-tests, which show that the average value is significantly higher than 5, 6, 7, 7.5 and 7.75. The test only fails for a value of 8. This is a remarkable result, since the overall usefulness of this tool is considered higher than 7.75.

**Table 5:** One sample parametric t-tests for question 15

Test value	Statistic (t)	Significance	Difference between means
5.00	10.983	**< 10**^**–3**^	3.533
6.00	7.875	**< 10**^**–3**^	2.533
7.00	4.766	**< 10**^**–3**^	1.533
7.50	3.212	**.006**	1.033
7.75	2.435	**.029**	.783
8.00	1.658	.120	.533

Bold values highlight significant figures

## Conclusions

The role of RAS in surgical practice is becoming increasingly important. Therefore, it is also increasingly important to introduce this paradigm into surgical training programs. For this reason, this article presents an MR-based tool for the stereoscopic visualization, virtual annotation and collaborative display of RAS surgical procedures. Although there are some applications that allow the viewing of stereoscopic videos, there is no academic work that allows the annotation and display of RAS videos in the way our tool does. Our application is web-based, and therefore, almost universal. It can work both on local networks and also remotely. It works on computers, tablets and smartphones. More importantly, it can display MR-enhanced content about RAS procedures with both expensive (i.e., an HMD) and inexpensive hardware (e.g., a $1 VR Cardboard). It is also collaborative, since a teacher can share a class with several students. Finally, it allows virtual content, in the shape of virtual annotations, to be introduced into an existing stereoscopic video.

An experimental test with RAS students was performed with good results. None of the users had problems using the visualization device, and the researchers observed that all of them were pleasantly surprised with the proposed visualization system. The results obtained with the evaluation support this initial impression. Given these results obtained with the evaluation, we can confidently say that the tool is useful and helps train surgeons in RAS. All the questions received very positive answers. The items that were the most positively rated were the usefulness of 3D visualization to understand the position and orientation of the surgical material in relation to the patient's anatomical elements, and the use of sections to reduce the amount of video that needs to be watched. Some of the questions regarding the annotations received slightly worse responses, although none of the responses received an average value lower than 6 (in a 1–7 Likert scale). In addition, the users felt that the annotations were well integrated and well visualized within the video, since this question received a value significantly higher than 6. Regarding the limitations of the study, we acknowledge that this is a preliminary evaluation with 15 users. This is not a large number, but it is also true that this is a highly specific application and it is quite difficult to find a large group of people who could be considered potential users of this application. Our target group is small, since this tool is designed for surgeons who are enrolled on RAS courses. Therefore, it is unrealistic to expect to recruit a large group of such people. Nevertheless, it is also true that we performed the test in an uncontrolled non-local network—since the server and the clients of the application were on different networks—with wireless devices, and no communication problems were reported. This increases the validity of the assessment.

Future work includes the evaluation of the authoring tool with a usability or effort test, such as the SUS [51] or the NASA-TLX [52]. We should also research into improving the annotation system, since it is possible that in some situations the tracking system of the annotation process can fail to track the selected object for the desired amount of time. Depth perception could also be enhanced, but this improvement entails firstly obtaining accurate depth information about the pixels of the video, something that is not generally available at first hand. Finally, we expect to move our research to the next phase and test the amount of learning transference that we can achieve with this tool.
